# Optimization of erythritol production through fermentation using molasses as carbon source

**DOI:** 10.3389/abp.2024.14000

**Published:** 2025-01-08

**Authors:** Riahna Kembaren, Arli Aditya Parikesit, Jocelyn Nataniel, Nethania Angeline Dharmawan, Charlivo Mikaichi Dungus, Priscilla Angelique, Solmaz Aslanzadeh

**Affiliations:** Department of Biotechnology, Indonesia International Institute for Life Sciences, East Jakarta, Indonesia

**Keywords:** erythritol production, C/N ratio, NaCl, pH, fed-batch fermentation

## Abstract

Erythritol is a beneficial sugar alcohol that can be used as a sugar substitute for diabetic patients. Erythritol is a bioproduct produced by microorganisms as a response to high osmotic pressure and stress in the growth medium. High concentrations of carbon source substrate can increase the osmotic pressure and provide more nutrient supply for yeast growth and metabolism. Aside from that, an optimal carbon-to-nitrogen (C/N) ratio can also make the erythritol conversion pathway more favorable. Therefore, this research aims to determine the optimal concentrations of molasses as the carbon source, yeast extract as the nitrogen source, and the optimal carbon-to-nitrogen (C/N) ratio to achieve the highest erythritol productivity. The research also seeks to optimize NaCl concentrations and pH while comparing batch and fed-batch fermentation systems to determine which produces a higher erythritol yield. One-Factor-at-A-Time (OFAT) method was used to identify optimal production conditions. The study found that the highest erythritol concentration, 17.48 ± 0.86 g/L, was achieved using 200 g/L of molasses, 7 g/L of yeast extract (200/7), and 25 g/L of NaCl, with a yield mass of 0.262 ± 0.00 g/g and a volumetric productivity of 0.095 ± 0.021 g/Lh. The pH optimization revealed that the best erythritol production occurred within a pH of 5. Furthermore, fed-batch fermentation significantly increased erythritol concentration to 26.52 ± 1.61 g/L, with a yield mass of 0.501 ± 0.032 g/g and a volumetric productivity of 0.158 ± 0.01 g/Lh. These findings emphasize the importance of optimizing carbon source, nitrogen source and NaCl concentration, pH, and fermentation systems, particularly highlighting the benefits of fed-batch fermentation in maximizing erythritol production. These findings provide a solid foundation for improving erythritol yields for industrial applications.

## Introduction

Sweeteners have been regarded as a food additive widely used to impart a sweet taste to food and as a substitute for high-calorie sugars. One of the sweeteners included is sucrose, a common sweetener widely marketed, as it is cost-effective and available in many forms (Kar et al., 2019). However, despite the advantages it offers, sucrose poses health risks for people with diabetes mellitus, as it has a glycogenic property, and its high intake will be detrimental to humans (Biggelaar et al., 2017). Based on Sun et al. (2022) findings, globally, diabetes mellitus prevalence has been projected to be at 12.2% (783.2 million people) in 2045. Addressing this issue, the intake of low-calorie natural sweeteners has been arising. Erythritol, a polyol, has become a current trend as one of the most used natural sweeteners and alternatives to sucrose. Although erythritol offers a sweetness level similar to sucrose (70%–80%), it is lower than xylitol and maltitol (up to 90% compared to sucrose). However, erythritol has a significantly lower glycemic index than both xylitol and maltitol, making it a healthier alternative. Erythritol can be sourced from various fruits and fermented food products (Mazi and Stanhope, 2023).

Erythritol can be produced via chemical synthesis and hydrogenation process. However, it has yet to be preferred for commercial production due to the low yields and relatively high cost associated with its process (Rzechonek et al., 2018). A biotechnological approach can be one of the ways to produce erythritol. Erythritol can be produced as a bioproduct by microorganisms. As a defense mechanism product, erythritol is proven to accumulate more in high osmotic stress in the media during the pentose-phosphate pathway (PPP). High concentrations of carbon sources can elevate the osmotic pressure in the growth media (Yang et al., 2014; Rywińska et al., 2024a). However, excess osmotic pressure can negatively impact the yeast growth, leading to cell death, whereas excess carbon sources will be economically unfeasible. Consequently, optimization should be considered to improve erythritol production with optimum growth conditions. Using cheap carbon sources is beneficial for accomplishing the low-cost substrate erythritol production, which can be from industrial byproducts, such as molasses. As one of the alternative carbon sources, molasses is a byproduct of sugar production, attributed to dense–viscous liquid form and lack of water, mainly consisting of non-reducing and reducing sugars (sucrose, glucose, fructose), and minerals. As a byproduct, molasses can limit the carbon footprint (Seshadrinathan and Chakraborty, 2022; De Vasconcelos, 2015). For this reason, the bioconversion of molasses into erythritol is a promising practice for a circular economy as it reduces waste while creating a higher-value product.

Several variables are the key determining factors for erythritol production, including the carbon source, nitrogen source, osmotic pressure, and fermentation operation mode (Daza-Serna et al., 2021). As suggested by Hijosa-Valsero et al. (2022), the carbon-to-nitrogen (C/N) ratio is a pivotal factor to be adjusted for escalating the erythritol production in a balanced ratio, as the imbalance of C/N ratio may shift the metabolic pathway, lowering the carbon flux for erythritol production. As erythritol production occurs under stress conditions, the NaCl concentration and pH must also be optimized. Other than that, the mode of fermentation operation process, batch or fed-batch, is remarkably important due to each advantage and disadvantage this system has, which could increase erythritol yield. The batch fermentation system allows the addition of media in the initial fermentation, while the fed-batch fermentation system provides a gradual nutrient addition in the media within a specific period (Daza-Serna et al., 2021; Tomaszewska et al., 2014). The selection of which mode of fermentation operation will rely on the microorganisms chosen, the bioproduct, and the cost-effectiveness of the erythritol production.

Our preliminary research suggests that yeast *Moniliella polinis* mutant SP5, obtained from random mutagenesis using UV irradiation, was able to produce 13.59 ± 0.812 g/L of erythritol in fermentation media containing 200 g/L of molasses (carbon source), 1 g/L of yeast extract (nitrogen source), and 25 g/L of NaCl (osmotic pressure) after 7 days of fermentation. However, after 4 days, the glucose and sucrose concentration in molasses was nearly depleted, followed by a decrease in cell viability and termination of the erythritol accumulation. Following these results, the potential to elevate erythritol production by further optimization is promising. This research aims to determine the optimal concentrations of molasses as the carbon source, yeast extract as the nitrogen source, and the optimal carbon-to-nitrogen (C/N) ratio to achieve the highest erythritol productivity. Additionally, it aims to optimize NaCl concentration and pH while comparing batch and fed-batch fermentation systems to ascertain which method yields a higher erythritol output. This research hypothesizes that increased molasses concentration may lead to higher erythritol production, as higher levels of yeast extract, and that fed-batch fermentation may yield greater erythritol output. In this study, One-Factor-at-A-Time (OFAT) optimization was used. OFAT is an experimental design approach used to evaluate the impact of individual variables on outcomes, making it useful for optimizing erythritol production via microbial fermentation. In OFAT experiments, one variable is adjusted while keeping others constant to isolate its effects on erythritol yield, thereby identifying optimal conditions for production.

## Materials and methods

### Preparation of *Moniella pollinis* SP5 preculture

A colony of *M. pollinis* mutant SP5 was streaked using an inoculation loop and then cultivated in a 300 mL of Potato Dextrose Broth (PDB) media. The pre-culture was incubated in a shaker incubator with agitation of 120 rpm at 30°C temperature for 3 days.

### Preparation of molasses-yeast extract-mineral (MYM) media

The Molasses-Yeast Extract-Mineral (MYM) media was prepared by adding molasses, yeast extract, NaCl, 2 g/L of NH_4_Cl, 1 g/L of MgSO_4_.7H_2_O, and 0.2 g/L of KH_2_PO_4_ into an Elenmeyer flask. The components were then hydrated with type III water to a final volume of 300 mL. The solution was mixed, sealed with aluminum foil, and autoclaved at 121°C for 15 min.

### Design optimization of erythritol production using one-factor-at-A-time (OFAT)

**FIGURE 1:**
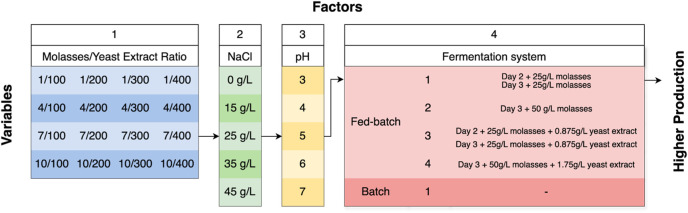
A Flowchart of the OFAT method for erythritol production optimization.

To identify the optimum value of each variable treatment, erythritol design optimization was conducted using a One-Factor-at-A-Time (OFAT) approach. The number of variables was reduced due to the screening with OFAT, selecting the best factor obtained. Four variables were tested: the Molasses/Yeast Extract ratio, NaCl concentration, pH, and the fermentation system. Each variable of each factor can be seen in Figure 1.

### Optimization of molasses-to-yeast extract ratio in the fermentation media

The molasses-to-yeast extract ratio optimization was done using MYM media with different molasses concentrations (100, 200, 300, and 400 g/L) and different yeast extract concentrations (1, 4, 7, and 10 g/L). In this study, molasses was used as the carbon source, while yeast extract served as the nitrogen source. *M. pollinis* SP5 pre-culture was inoculated with a volume of 10% (v/v) from the total volume of MYM media in the flask and was then grown at a temperature of 30°C and 120 rpm for 7 days. Aliquot of the culture was taken daily for growth analysis, as well as erythritol production and molasses consumption analysis using High-Performance Liquid Chromatography (HPLC). These procedures were done in biological duplicate and technical triplicate.

### Optimization of NaCl concentration in the fermentation media

After determining the optimum molasses-to-yeast extract concentration ratio, we optimized the MYM media with different NaCl concentrations (0 g/L, 15 g/L, 25 g/L, 35 g/L, and 45 g/L). *M. pollinis* SP5 pre-culture was inoculated with a volume of 10% (v/v) from the total volume of MYM media in the flask and was then grown at a temperature of 30°C and 120 rpm for 7 days. Aliquot of the culture was taken daily for growth analysis, as well as erythritol production and molasses consumption analysis using HPLC. These procedures were done in biological (fermentation) duplicate and technical triplicate.

### Optimization of initial pH in the fermentation media

After obtaining the optimum molasses-to-yeast extract concentration ratio and NaCl concentration, the pH optimization was done using MYM media with different initial pH levels (3, 4, 5, 6, and 7) that were adjusted using HCl or NaOH. A pH of 5 serves as the control, representing the natural pH of the MYM media. *Moniliella pollinis* SP5 preculture was inoculated with a volume of 10% (v/v) from the total volume of MYM media in the flask and was then grown at a temperature of 30°C and 120 rpm for 7 days. Aliquot of the culture was taken daily for growth analysis, as well as erythritol production and molasses consumption analysis using HPLC. These procedures were done in biological duplicate and technical triplicate.

### Optimization of fermentation operation system (batch and fed-batch)

*M. pollinis* SP5 pre-culture was inoculated with a volume of 10% (v/v) from the total volume of MYM media in the flask and was then grown at a temperature of 30°C and 120 rpm for 7 days. Four different feeding treatments were done for fed-batch fermentation by adding nutrients to the fermentation media. For the first treatment, 25 g/L of molasses was added on the second and third day of incubation. For the second treatment, 50 g/L of molasses was added only on the third day. For the third treatment, 25 g/L of molasses and 0.875 g/L of yeast extract were added on the second and third days. For the fourth treatment, 50 g/L of molasses and 1.75 g/L of yeast extract were added only on the third day. Aliquot of the culture was taken daily for growth analysis, as well as erythritol production and molasses consumption analysis using HPLC. These procedures were done in biological duplicate and technical triplicate.

### Growth kinetic analysis using colony forming unit (CFU) measurement

Each aliquot of fermentation broth sample was diluted with a ten-fold dilution by adding 900 µL PBS to the microtubes, and then 100 µL of the sample was taken to each dilution. It was then continued with the same serial dilution pattern until the last dilution factor.

Potato Dextrose Agar (PDA) plates were divided into four sections and labeled with the four smallest dilution factors. Three drops of the dilution, each 10 μL, were dropped into each section. Once the droplets had dried, the plates were closed and sealed with parafilm. The plates were then turned upside down and incubated at room temperature for 4–5 days before the colony was counted. Then, the colony-forming unit per milliliter (CFU/mL) was calculated.

This measurement was done daily until 7 days of fermentation (T7). At the end of the incubation period, the graph was plotted using GraphPad Prism 8.0.1.244 (United States) with the *x*-axis as the time (days) versus the *y*-axis of log CFU/mL to generate a growth curve of *M. pollinis* SP5.

### Dry cell weight measurement and supernatant collection

An empty microcentrifuge tube was first weighed before 1 mL of the aliquot fermentation broth sample was added. The tube was then centrifuged at 5,000 rpm for 15 min. The supernatant was collected and transferred to a new microcentrifuge tube using a micropipette and stored in the fridge for HPLC analysis. The cell pellet was left inside an oven at 60°C for 2 days. The dry cell weight was then calculated by subtracting the weight of empty microcentrifuge tubes and total dried cell pellets with microcentrifuge tubes. At the end of the incubation period, the graph was plotted using GraphPad Prism 8.0.1.244 (United States) with the *x*-axis as the time (days) versus the *y*-axis of g/L dry cell weight to generate a dry cell weight curve of *M. pollinis* SP5.

### pH level measurements in the fermentation media

A total of 3 mL sample was collected in a 15 mL falcon tube. A pH meter (OHAUS 3100, United States) was then used to measure the pH level in triplicate. At the end of the incubation period, the graph was plotted using GraphPad with the *x*-axis as the time (days) versus the *y*-axis of pH value to generate a pH curve during the fermentation process.

### Quantification of sugar concentration

Erythritol standard solutions with various concentrations (1.25, 2.5, 5, 10, and 20 mg/mL) were prepared for the standard curve. The same was done for the molasses’ sugar, which includes glucose, fructose, and sucrose (12.5, 25, 50, 50, 100, and 200 mg/mL). Both standards and samples were inserted into HPLC vials using a syringe through a 0.22 μm polyethersulfone membrane filter from Minisart^®^. All vials were then inserted into the HPLC machine.

HPLC (Thermo Scientific Dionex UltiMate 3000 HPLC) with Shodex SUGAR SP0810 column (8.0 mm I.D. × 300 mm) as the sugar analytical column was used to quantify the erythritol production and molasses’ sugar content, especially glucose, sucrose, and fructose consumption. The HPLC running was conducted using the settings of an upper limit at 50 bars, a column temperature at 70°C, a mobile phase of 100% filtered type 1 water, a refractor index (RI) detector at 50°C, a flow rate of 0.7 mL/min, and 30 min processing duration. The HPLC results were used to measure the molasses’ sugar content consumption and erythritol concentration within the fermentation broth samples.

### Calculation of fermentation parameters

Erythritol production was evaluated based on the yield mass of erythritol (Y_ery_) and the volumetric productivity of erythritol (Q_ery_) (Rakicka-Pustułka et al., 2020). The yield mass of erythritol (Y_ery_) (g/g) was calculated using the following formula (Equation 1):

$$Y_{\text{ery}}=\frac{P}{S}$$ (1)

The volumetric productivity of erythritol (Q_ery_) (g/Lh) is calculated using the formula (Equation 2):

$$Q_{\text{ery}}=\frac{P}{V\;t}$$ (2)

where *P* is the erythritol product (g), *S* is the substrate consumed (g), *V* is the culture volume (L), and *t* is the fermentation time (h).

### Statistical analysis

An unpaired t-test was conducted for the replicates using the GraphPad Prism program Ver. 8.0.1 (GraphPad Software, Inc., California, United States) for the sugar consumption and erythritol production data. Data are expressed as the average of the replicates ± standard deviation (SD). The differences were considered low and high statistically significant at probability *p* < 0.05 and 0.01, correspondingly.

## Results and discussion

### Optimization of molasses-to-yeast extract ratio

One of the key factors influencing erythritol production is the used of substrate ratio, which in this experiment is the molasses-to-yeast extract concentration ratio in the fermentation media. The balance between these nutrients plays an essential role in regulating microbial metabolism, directly affecting the efficiency of erythritol biosynthesis pathways. High molasses concentrations provide the necessary substrates for erythritol production, while yeast extract as nitrogen sources are vital for cell growth and enzyme production. However, an imbalance in the molasses-to-yeast extract concentration ratio can lead to suboptimal erythritol yields due to the diversion of metabolic activity towards cell growth or other byproducts (Anzola-Rojas et al., 2015; Hijosa-Valsero et al., 2022). The experiment was conducted by dividing the media combination according to its yeast extract concentration; starting from 1, 4, 7, and 10 g/L; with all the molasses concentrations, which include 100, 200, 300, and 400 g/L.

**FIGURE 2:**
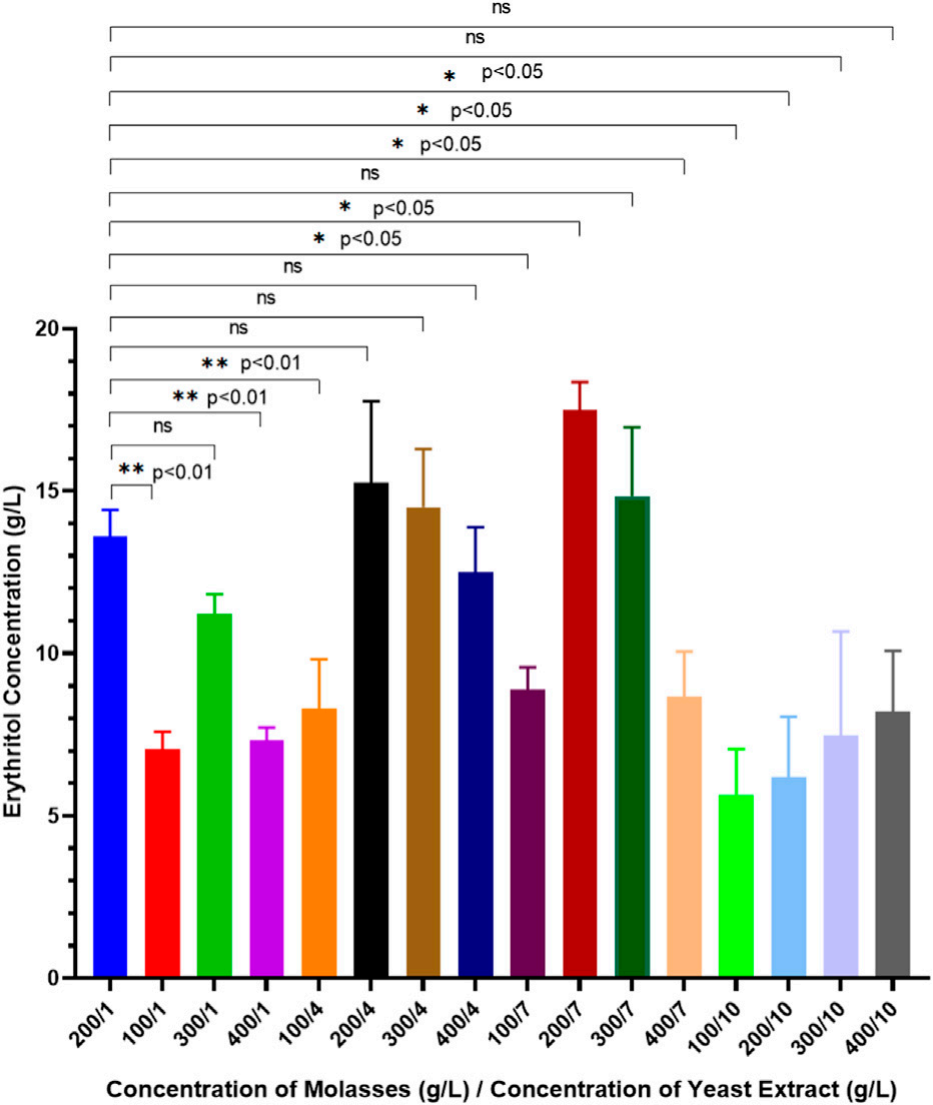
Erythritol production by *Moniliella pollinis* SP5 with different concentrations of molasses and yeast extract in the MYM fermentation media.

Figure 2 shows erythritol concentrations after 7 days of fermentation using *M. pollinis* SP5 with different molasses and yeast extract concentration combinations. Results indicated that the highest erythritol concentration of 17.48 ± 0.86 g/L was obtained when *M. pollinis* SP5 was grown in MYM media with 200 g/L of molasses and 7 g/L of yeast extract. Erythritol production using 100 g/L molasses with all combinations of yeast extract produced the lowest erythritol concentration compared to other molasses concentrations. This is due to the insufficient amount of carbon provided by 100 g/L molasses for *M. pollinis* growth and also low osmotic pressure, which causes low erythritol production. However, using higher carbon and nitrogen concentrations is not necessarily related to higher erythritol production, as the carbon flux within the cells during fermentation might lead to the production of biomass of cells and byproducts. Erythritol production using 400 g/L of molasses with all combinations of yeast extracts produced the second lowest erythritol concentration compared to other molasses concentrations (Figure 2). Erythritol synthesis is proportional to carbon flux through the pentose-phosphate pathway (PPP) and is regulated by oxidative and non-oxidative PPP enzymes. Erythritol can be synthesized from glucose in molasses through the PPP. Glucose-6-phosphate dehydrogenase (G6PD) is a rate-limiting enzyme of the PPP. The abundance of NADPH within the cell when cultivated in an extremely high carbon source concentration has a feedback inhibition on the G6PD enzyme and inhibits the PPP metabolism pathway, which causes a decrease in the erythritol production (Ortiz et al., 2022). Moreover, excess molasses also adds as a source of osmotic stress in cell culture. In conditions with extremely high osmotic stress, the cell will alter the production towards more effective endogenous osmolytes compared to erythritol, such as sorbitol, to combat hyperosmotic stress. This condition could also explain the decrease in erythritol production (Ortiz et al., 2022).

Erythritol production was further evaluated by calculating the yield mass (g/g) and volumetric productivity (g/Lh) in all molasses and yeast extract combinations of fermentation media using *M. pollinis* SP5 as the cell factory. As previous research initially used 200 g/L of molasses and 1 g/L of yeast extract (200/1), this combination acts as the control in order to determine the significant difference in erythritol production using unpaired t-test (*p* < 0.05), with other MYM media combinations. From the unpaired Student’s t-test analysis, a significant increase in erythritol concentration was achieved using a media combination of 200 g/L molasses and 7 g/L yeast extract (200/7) with a yield mass of 0.262 ± 0.00 g/g, and volumetric productivity of 0.095 ± 0.021 g/Lh (Table 1). These results indicate that production using 200 g/L molasses and 7 g/L yeast extract is the most optimal concentration ratio, where the concentration of carbon and nitrogen sources is sufficient for cell growth, to provide enough osmotic pressure to stimulate erythritol without negative effects, and the optimal carbon-to-nitrogen ratio for enzyme activity in synthesizing erythritol. During the fermentation period using *M. pollinis* SP5 from day 0 to day 7 of fermentation, no significant pH changes were observed in all combinations of fermentation media. The pH of the media was maintained (Supplementary Figure S1). For fermentation media using 10 g/L of yeast extract, the pH was the highest, which was pH 6 (Supplementary Figure S1). High concentrations of yeast extract as a nitrogen source increased the media’s pH, making it more alkaline compared to media with lower yeast extract levels. However, during fermentation, this pH was still maintained even though it produced organic acids such as citric acid (Bellou et al., 2014). This is due to the pH buffer capacity of molasses (Rzechonek et al., 2018).

**TABLE 1:** Productivity of *M. pollinis* SP5 to produce erythritol using different compositions of molasses and yeast extract in the fermentation media.

Media combination (molasses/yeast extract) (g/L)	Erythritol concentration (g/L)	Erythritol yield mass (g/g)	Erythritol volumetric productivity (g/Lh)	Comparison to control
100/1	7.05 ± 0.53	0.185 ± 0.04	0.057 ± 0.017	** (decrease)
200/1	13.59 ± 0.812	0.181 ± 0.04	0.061 ± 0.020	Control
300/1	11.21 ± 0.59	0.099 ± 0.01	0.074 ± 0.016	ns (decrease)
400/1	7.33 ± 0.39	0.077 ± 0.00	0.044 ± 0.003	** (decrease)
100/4	8.29 ± 1.52	0.184 ± 0.01	0.049 ± 0.013	** (decrease)
200/4	15.24 ± 2.51	0.179 ± 0.01	0.080 ± 0.036	ns (increase)
300/4	14.49 ± 1.79	0.173 ± 0.05	0.073 ± 0.002	ns (increase)
400/4	12.48 ± 1.39	0.164 ± 0.00	0.060 ± 0.023	ns (decrease)
100/7	8.87 ± 0.69	0.225 ± 0.03	0.058 ± 0.010	* (decrease)
200/7	17.48 ± 0.86	0.262 ± 0.00	0.095 ± 0.021	* (increase)
300/7	14.83 ± 2.12	0.257 ± 0.01	0.088 ± 0.018	ns (increase)
400/7	8.66 ± 1.38	0.151 ± 0.06	0.053 ± 0.011	* (decrease)
100/10	5.65 ± 1.40	0.069 ± 0.047	0.033 ± 0.012	* (decrease)
200/10	6.18 ± 1.87	0.062 ± 0.053	0.036 ± 0.015	* (decrease)
300/10	7.48 ± 3.18	0.057 ± 0.039	0.044 ± 0.0026	ns (decrease)
400/10	8.12 ± 1.87	0.066 ± 0.059	0.048 ± 0.015	ns (decrease)

Note: “ns” indicates no significant difference (*p* > 0.05), “*” indicates a significant difference (*p* ≤ 0.05) “**” indicates a more significant difference (*p* ≤ 0.01) toward the experimental controls.

**FIGURE 3:**
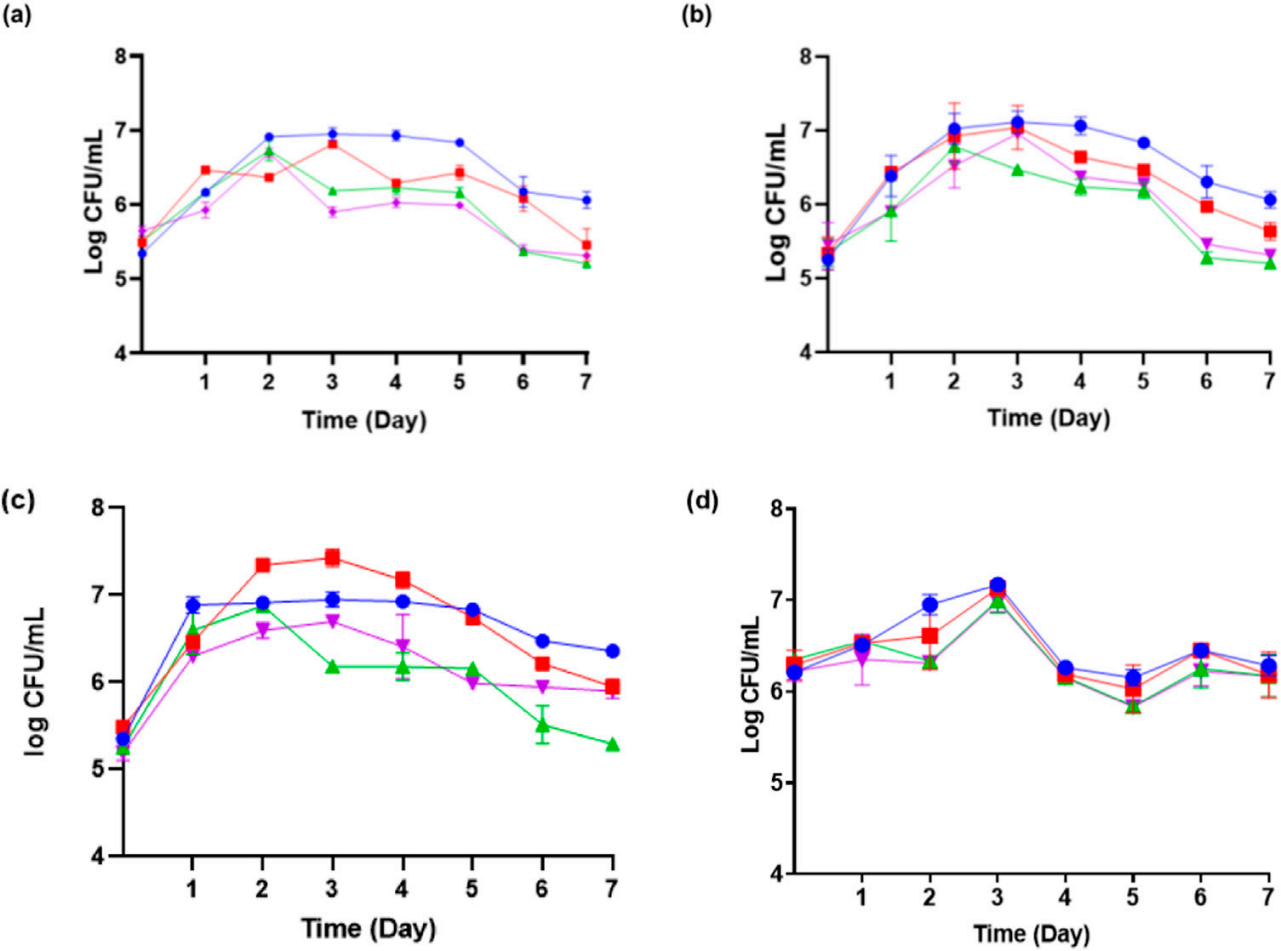
The growth kinetic of *M. pollinis* SP5 on fermentation media with different molasses-to-yeast extract concentration ratios using **(A)** 1 g/L; **(B)** 4 g/L; **(C)** 7 g/L; and **(D)** 10 g/L of yeast extract in combination with different molasses concentrations: 100 g/L (blue), 200 g/L (red), 300 g/L (green), and 400 g/L (purple).

*Moniliella pollinis* SP5 has an invertase enzyme that can hydrolyze sucrose into fructose and glucose, which can then be used by *M. pollinis* SP5. Upon observation of *M. pollinis* SP5 growth phase in all MYM media combinations, it was seen that the cells went through the log phase up until the second to third day of incubation, continued with the stationary phase until the fifth day, and finally entered the death phase from the sixth day in all media (Figure 3). Erythritol is produced by *M. pollinis* SP5 in the stationary phase (Deshpande et al., 2022). In the fermentation media containing 1, 4, and 7 g/L of yeast extract, the viable cell count is lower with 400 g/L of molasses compared to 100 g/L of molasses. The results indicate that higher carbon concentrations increase osmotic pressure, which inhibits the proliferation of *M. pollinis* SP5. However, this trend is not observed in MYM media with 10 g/L yeast extract, which might be due to the bigger osmotic pressure and non-optimal substrate ratio due to the relatively high amount of yeast extract in the fermentation media (Figure 3D).

Daily dry cell weight measurements were also performed to monitor the growth of the *M. pollinis* SP5 culture in the flask (Supplementary Figure S2). The results show an increase in dry cell weight throughout the experiment. Cell biomass production was observed to increase more rapidly during the first half of fermentation, with a significant drop in sugar concentration and active yeast growth (log phase) from the start of cultivation until the third day. Growth began to slightly increase to plateau, after the third day as the viable cell number decreased. The increase in dry cell weight is primarily influenced by the rate of cell growth and the cells’ ability to utilize nutrient sources in the medium. The availability of nutrients for metabolic activities and a higher nutrient concentration promote increased cell numbers during incubation (Malairuang et al., 2020).

### Optimization of NaCl concentration in the fermentation media

One of the critical factors affecting erythritol production is the concentration of sodium chloride (NaCl) in the fermentation media. NaCl plays a role in microbial metabolism, primarily by creating osmotic pressure that can influence the stress response of microorganisms. In high-osmotic-pressure environments, many microorganisms switch to stress response pathways, which can lead to enhanced production of certain metabolites like erythritol. NaCl concentration, therefore, directly impacts the cell’s physiological state. In erythritol biosynthesis, applying the appropriate NaCl concentration can significantly boost the conversion of carbon sources into erythritol by inducing a hyperosmotic environment that favors the pathway for polyol (sugar alcohol) production (Ortiz et al., 2022). However, excessive NaCl concentrations may inhibit cell growth and reduce erythritol yield, making it crucial to identify an optimal concentration that balances stress without harming the microorganisms (Carly and Fickers, 2018). Moreover, excessive NaCl in fermentation media could disrupt cellular osmoregulation, leading to dehydration or ion toxicity (Li et al., 2021).

**FIGURE 4:**
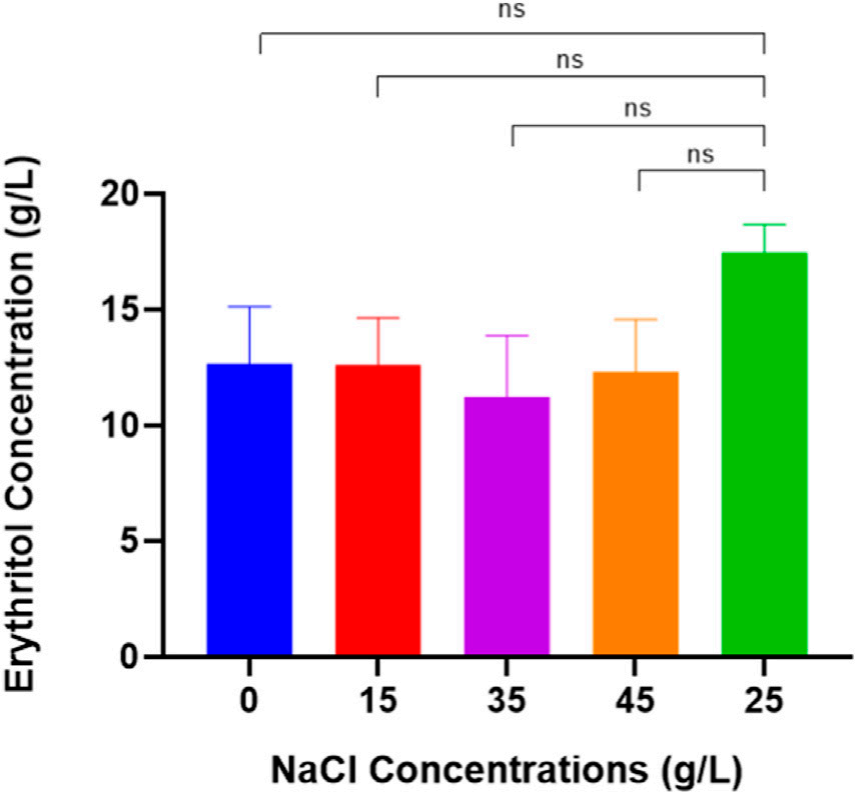
Erythritol Production by *Moniliella pollinis* SP5 using different concentrations of NaCl: 0 g/L (blue), 15 g/L (red), 35 g/L (purple), 45 g/L (orange), and 25 g/L (green) for control.

Based on the HPLC results, the highest concentration of erythritol produced by *M. pollinis* SP5 when grown on MYM media with a NaCl concentration of 25 g/L compared to other NaCl concentrations in the fermentation media, which produced erythritol of 17.48 ± 0.86 g/L (Figure 4). At a concentration of 25 g/L, the salt concentration provides an optimal osmotic stress to increase the production of polyol components such as erythritol (Rzechonek et al., 2018). Based on previous research with other types of osmophilic yeast, Yang et al. (2015) used *Yarrowia lipolytica* CICC 1675 to produce erythritol; they found NaCl 30 g/L with 4.15 osmol/kg can produce 98.5 g/L of erythritol. Moreover, another study by Yang et al. (2024), found that adding 25 g/L NaCl can increase the production of erythritol by about 19.3% using *Y. lipolytica* E326 (a strain derived from *Y. lipolytica* PO1F). By adding NaCl, it can make osmophilic yeast like *Moniliella* sp.*, Candida* sp., or *Yarrowia* sp. live in a hypertonic environment and boost carbon flows to the pentose phosphate pathway (Liu et al., 2022). Overly high and low concentrations of NaCl can decrease desirable polyol (erythritol) synthesis (Mirończuk et al., 2015).

**FIGURE 5:**
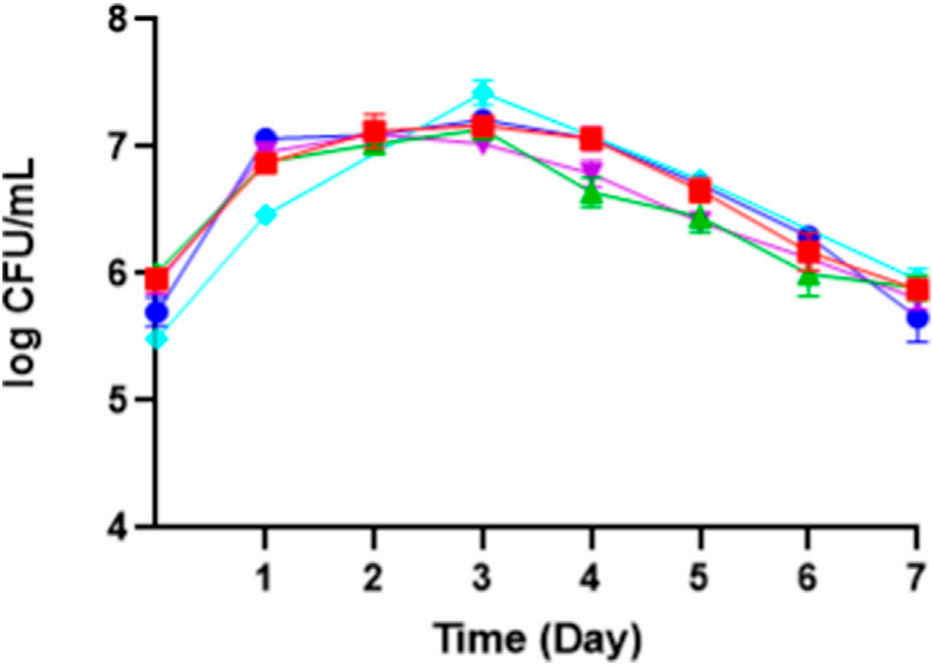
The growth phase of *M. pollinis* SP5 on different NaCl concentrations: 0 g/L (blue), 15 g/L (red), 25 g/L (control) (turquoise), 35 g/L (green), and 45 g/L (purple).

According to Table 2, the highest yield mass and volumetric productivity of erythritol were recorded at 0.262 ± 0.00 g/g and 0.095 ± 0.021 g/L·h, respectively, using a NaCl concentration of 25 g/L. The data trend indicates that NaCl concentrations of 0 and 15 g/L resulted in lower erythritol yields compared to the 25 g/L concentration. However, this decrease was not statistically significant relative to the control (25 g/L), likely due to the sufficient osmotic pressure provided by the higher concentrations of molasses and yeast extract. Interestingly, even in the absence of NaCl, *M. pollinis* SP5 was capable of producing erythritol, supported by the osmotic pressure from 200 g/L of molasses and 7 g/L of yeast extract. Conversely, media containing 35 g/L and 45 g/L of NaCl showed reduced erythritol concentrations compared to the control (25 g/L). While this decrease was also not significantly different from the control. Figure 5 illustrates a slight inhibition of cell growth at higher NaCl concentrations: 7.03 log CFU/mL at 45 g/L and 7.14 log CFU/mL at 35 g/L, both lower than the control value of 7.43 log CFU/mL at 25 g/L. This growth inhibition may contribute to a reduced erythritol yield due to excessive osmotic stress.

**TABLE 2:** Erythritol concentration (g/L), erythritol mass yield (g/g), and erythritol volumetric productivity (g/Lh) from optimizing NaCl concentration of molasses-to-yeast extract ratio 200/7 in the fermentation media.

NaCl concentration (g/L)	Erythritol concentration (g/L)	Erythritol yield mass (g/g)	Erythritol volumetric productivity (g/Lh)	Comparison to control
0	12.66 ± 2.46	0.162 ± 0.018	0.075 ± 0.014	ns (decrease)
15	12.60 ± 2.04	0.184 ± 0.036	0.075 ± 0.012	ns (decrease)
25	17.48 ± 0.86	0.262 ± 0.00	0.095 ± 0.021	control
35	11.24 ± 2.63	0.222 ± 0.084	0.067 ± 0.015	ns (decrease)
45	12.31 ± 2.26	0.201 ± 0.072	0.073 ± 0.013	ns (decrease)

Note: “ns” indicates a not significant difference (*p* > 0.05) in relation to the experimental controls.

In addition, high erythritol production can be seen from the aspect of growth analysis such as colony forming unit (CFU) measurement (Figure 5), dry cell weight (Supplementary Figure S3), and pH (Supplementary Figure S4). Based on Figure 5, cell viability without NaCl addition indicates steady growth with minimal fluctuations, suggesting low dehydration and a stable metabolic state in the culture (Dupont et al., 2010). Despite that, the specific threshold of hydrostatic level was not examined in this experiment. The viable cell number in all NaCl concentrations used in this experiment exhibits a trend of gradual increase until day 3 and starts to decrease from day 5–7, dropping to around 6 log CFU/mL. The number of viable cells without NaCl addition shows a similar trend to that with NaCl addition. It indicates that the *M. pollinis* SP5 was able to tolerate NaCl until 45 g/L, and the cell still grew and did not reach a lethal NaCl concentration, in which cell death might occur. The study by Guyot et al. (2021) showed that *S. cerevisiae* CBS1171 can tolerate osmotic pressure up to a certain threshold, beyond which approximately 100 g/L of NaCl causes cell death due to extreme dehydration.

This study shows that in terms of biomass, the dry cell weight is closely related to CFU which increases until day 3 and remains stable until the seventh day. The pH of the media with the addition of NaCl and without addition of NaCL shows insignificant changes from day 0 to day 7 incubation, stable at around pH 4.5 to 5 level (Supplementary Figure S4). The presence of phosphate and the addition of salt from the media can also help in stabilizing the pH of the fermentation media (Wang et al., 2021).

### Optimization initial pH of the fermentation media

One of the critical parameters in the fermentation process is the initial pH of the growth medium. The pH level plays a pivotal role in microbial activity, influencing enzyme function and overall metabolic pathways. Additionally, the pH of the media can influence osmotic pressure, nutrient uptake, and microbial stress responses, all of which affect erythritol biosynthesis. For erythritol production, maintaining an optimal initial pH is essential for ensuring that the microorganisms involved in the fermentation process function efficiently (Mirończuk et al., 2015). A pH that is overly low or high can reduce the activity of key enzymes involved in the conversion of carbon sources into erythritol, thus limiting the overall yield. On the other hand, maintaining a pH range that supports the balance between microbial growth and erythritol synthesis can significantly enhance production efficiency.

**FIGURE 6:**
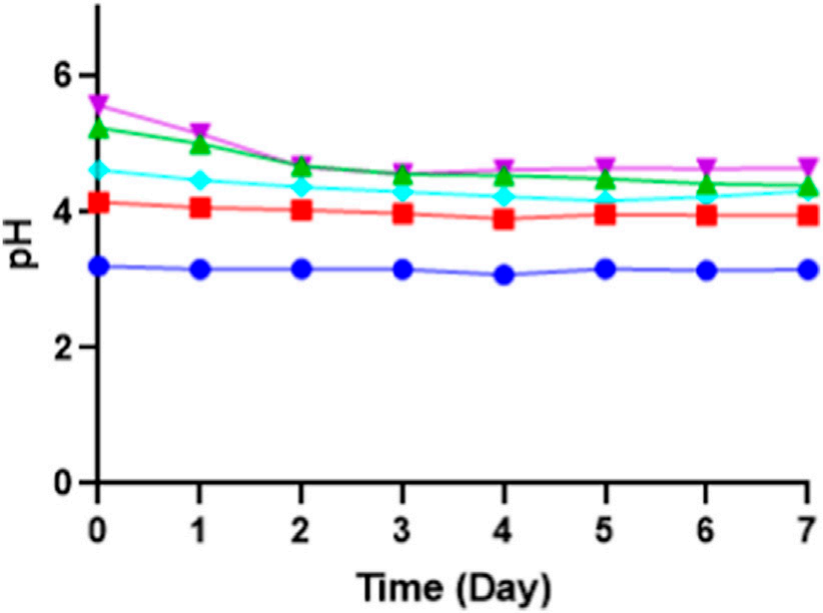
The pH of fermentation media for 7 days of fermentation on different initial pH of media: pH 3 (blue), pH 4 (red), pH 5 (control) (turquoise), pH 6 (green), and pH 7 (purple).

During the initial pH optimization, the media pH was adjusted from 3 to 7 before sterilization. Changes in pH after the sterilization were observed, particularly for pH 6 and 7. The pH 6 after autoclave dropped to 5.3, while pH 7 dropped to 5.6. Higher sugar content in the media further lowers the pH post-sterilization. This variation is due to different reactions among medium components during autoclaving; inorganic salts, amino acids, and vitamins all contribute to pH shifts. At higher temperatures, the increase in free ions in the solution results in lower pH values. However, when the initial pH is below 6, the pH change after autoclaving is less pronounced. Significant pH changes are observed primarily within the initial pH range of 5.7–8.5 (Skirvin et al., 1986; Chen et al., 2015). pH 3, 4, and 5 (control) tend to be stable even though autoclaved. The pH of the media can be seen in Figure 6. Fermentation for pH 3 has an initial pH around 3 and is steady and stable at pH 3, as well as pH 4 and pH 5 (control). However, with fermentation at an initial adjustment of pH 6, the pH drops from 5.3 to 4.4 after 7 days of fermentation, while with fermentation at an initial adjustment of pH 7, the pH drops from 5.6 to 4.6 after 7 days of fermentation. This drop in pH showed more organic acid production in the fermentation media when the initial pH was adjusted above pH 5.

**FIGURE 7:**
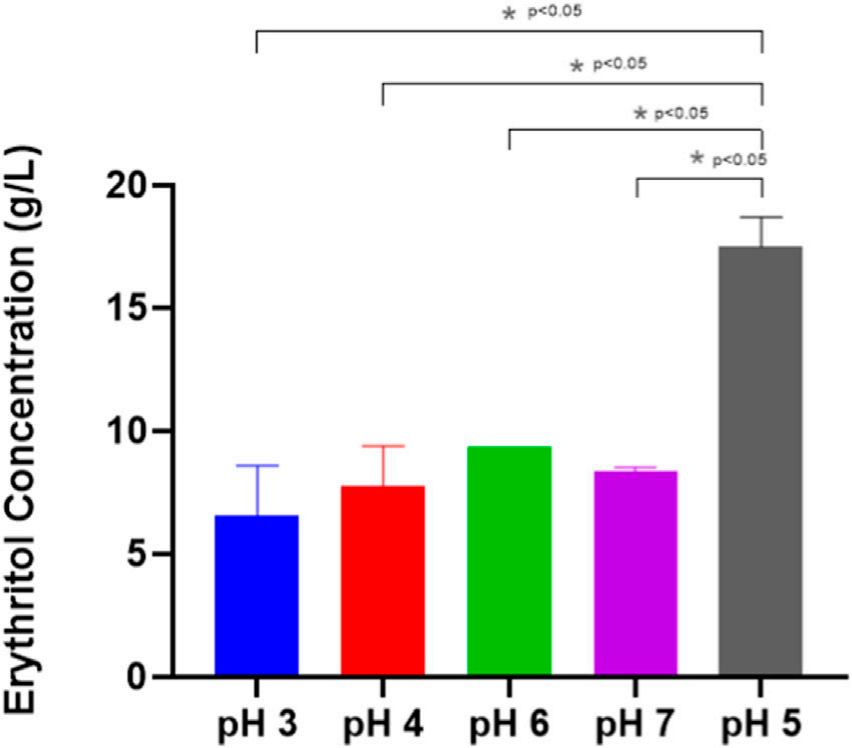
Erythritol Production by *Moniliella pollinis* SP5 at different initial pH of the fermentation media: pH 3 (blue), pH 4 (red), pH 5 (gray), pH 6 (green), and pH 7 (purple).

**FIGURE 8:**
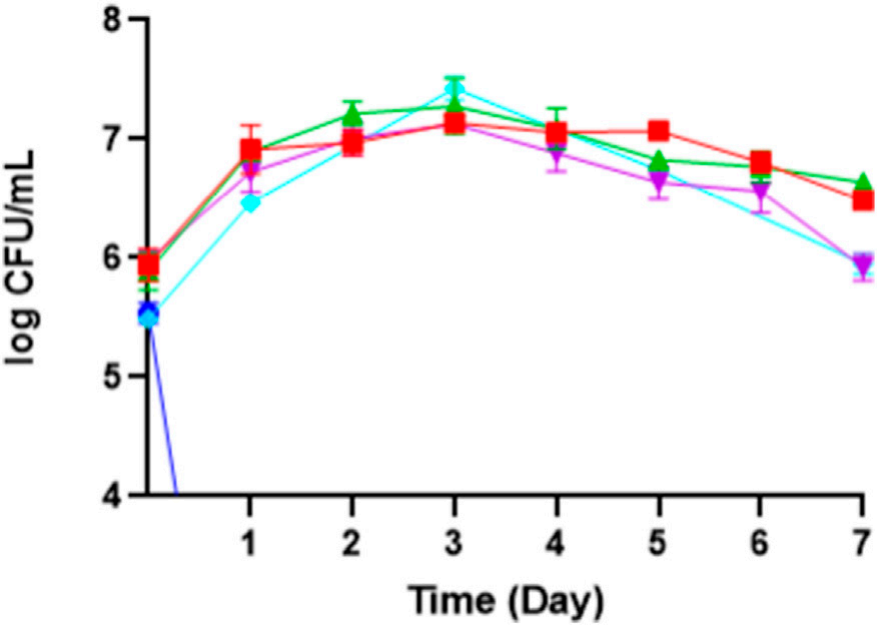
The growth phase of *M. pollinis* SP5 on different initial pH of fermentation media: pH 3 (blue), pH 4 (red), pH 5 (control) (turquoise), pH 6 (green), and pH 7 (purple).

This experiment found that the optimum pH for producing erythritol is around 5 (Figure 7), achieving 17.48 ± 0.86 g/L, and it is linear to the findings by Khatape et al. (2023), in which a mutated *M. pollinis* strain (mutant-58) can produce higher erythritol in pH 5.5 (92.2 ± 2.3 g/L), compared to the other pH treatments. pH levels outside the optimal range can disrupt the biosynthesis of erythritol, disrupting the internal pH, diverting the metabolic activity towards other byproducts, and hindering cell growth and productivity (Lund et al., 2020). This is proven by the decreasing of CFU and biomass at pH 3, where *M. pollinis* SP5 cannot survive at initial pH 3, even on day 1 (Figure 8). In the fermentation media with initial pH levels of 6 and 7, erythritol production was lower, likely due to increased organic acid byproducts such as citric, isocitric, succinic, itaconic, and acetic acids (Fickers et al., 2020), as evidenced by a more pronounced pH drop in those pH levels. Additionally, early pH shifts may have led to rapid adaptation demands on the cells, potentially causing metabolic inefficiencies and reducing the sugar conversion rate throughout fermentation (Mohd-Zaki et al., 2016; Li et al., 2020). The optimal pH for erythritol production varies depending on the specific microorganism used and the fermentation conditions. Different species can also be suitable to acidic conditions like at pH 3 can induce *Y. lipolytica* Wratislavia K1 strain to produce higher erythritol production of 40.7 g/L (Tomaszewska et al., 2014).

Based on Table 3, the highest mass yield and volumetric productivity of erythritol were 0.262 ± 0.00 g/g and 0.095 ± 0.02 g/Lh, respectively, at initial pH 5. The trend of data shows that at the initial pH lower or higher than pH 5, the erythritol concentration decreases. The lowest erythritol production is shown at an initial pH of 3 due to acid-induced cell death. The standard pH of MYM media (pH 5) is optimal for the growth of *M. pollinis* SP5, as it supports a greater increase in viable cell numbers during the log phase (Figure 8). A study conducted by Ghezelbash et al. (2012) reveals that a pH around 5.5 exhibits the highest polyols production, which aligns with the most optimum pH obtained in this study. The statistical analysis for the pH optimization significance is attached in Supplementary Table S3.

**TABLE 3:** Erythritol concentration (g/L), erythritol yield mass (g/g), and erythritol volumetric productivity (g/Lh) from initial pH optimization results with molasses/yeast extract of 200/7 ratio in the fermentation media.

Initial pH Treatment	Erythritol concentration (g/L)	Erythritol yield mass (g/g)	Erythritol volumetric productivity (g/Lh)	Comparison to control (*p*-value)
pH 3	6.58 ± 2.01	0.142 ± 0.01	0.040 ± 0.01	*(decrease)
pH 4	7.79 ± 1.61	0.120 ± 0.03	0.046 ± 0.01	*(decrease)
pH 5	17.48 ± 0.86	0.262 ± 0.00	0.095 ± 0.02	Control
pH 6	9.39 ± 0.00	0.089 ± 0.01	0.056 ± 0.00	*(decrease)
pH 7	8.36 ± 0.16	0.079 ± 0.00	0.049 ± 0.00	*(decrease)

Note: “*” indicates a significant difference (*p* > 0.05) toward the experimental controls.

### Optimization of the fermentation operation system (batch and fed-batch)

Optimizing the fermentation process is key to improve erythritol production. Batch fermentation involves adding all substrates at the start, but nutrient depletion and product inhibition can limit erythritol yield. In contrast, fed-batch fermentation gradually feeds substrates, offering better control over nutrients and reducing inhibition, thus potentially increasing yields. While fed-batch systems support higher cell density and prolonged production, they require careful optimization due to added complexity and cost (Rzechonek et al., 2018; Poontawee and Limtong, 2020). The optimization of erythritol production using the fed-batch mode of cultivation was then incorporated in this study. Fed-batch fermentation of *M. pollinis* SP5 was conducted for 7 days, similar to batch fermentation, yet the addition of substrate into the fermentation on specific days, depending on the treatments, as stated in the methods section.

**FIGURE 9:**
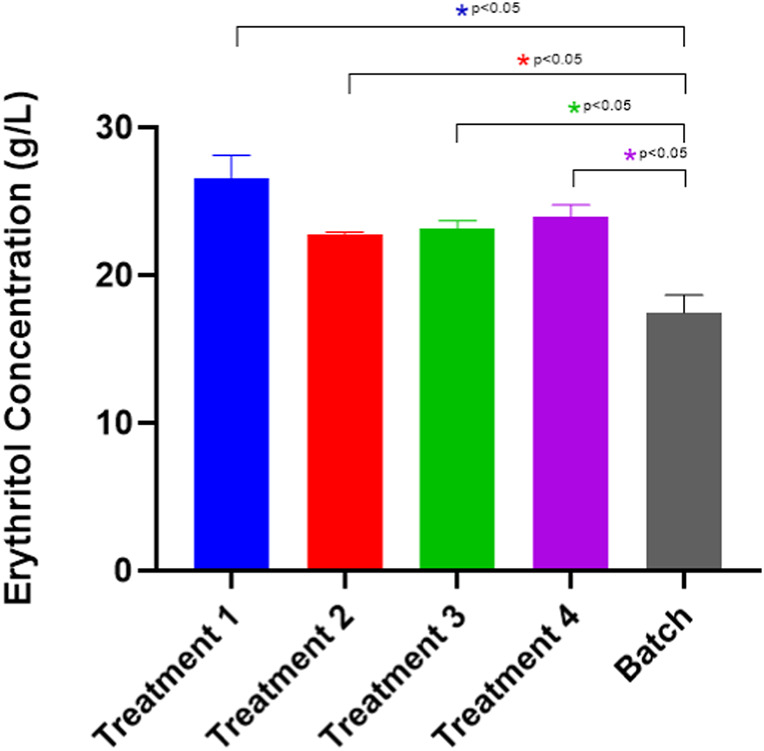
Erythritol production by *Moniliella pollinis* SP5 using batch and fed-batch fermentation systems with different feeding treatments. Treatment 1: Add 25 g/L of molasses on day-2 and day-3. Treatment 2: Add 50 g/L of molasses on day 3. Treatment 3: Add 25 g/L of molasses and 0.875 g/L of yeast extract on day-2 and day-3. Treatment 4: Add 50 g/L of molasses and 1.75 g/L of yeast extract.

Figure 9 depicts the erythritol production in the fed-batch mode of cultivation. All of the treatments exhibit significant increases compared to the control (batch). The first treatment was proven to accumulate the most erythritol with 26.52 ± 1.61 g/L. This result suggests that feeding twice on the second and the third day, adding only molasses at 25 g/L of concentration as the carbon source is the most optimum treatment to achieve a higher erythritol production. Based on the results, feeding twice on the second and third days with a lower substrate concentration (25 g/L) proves more optimal for erythritol production than a single feed at a higher concentration (50 g/L). Gradual substrate feeding allows cells to adapt more effectively to osmotic changes, avoiding the stress associated with abrupt increases. Additionally, split feeding supports stable enzyme activities within the pentose phosphate pathway (PPP), with the oxidative phase of the PPP remaining the primary route for erythritol synthesis without overloading the system (Rywińska et al., 2024b). The statistical analysis for the fed-batch optimization significance is attached in Supplementary Table S4.

Furthermore, as shown in Table 4, the highest erythritol production was found at the first treatment, with a yield mass and volumetric productivity of 0.501 ± 0.031 g/g and 0.158 ± 0.01 g/Lh, respectively. Aside from the ability of the first treatment to produce the highest erythritol concentration, the amount of substrate required was also the lowest. Therefore it was preferable, in terms of cost-effectiveness, compared to the other treatments. The erythritol production with the first treatment (26.52 ± 1.61) was also found to be significantly higher compared to the batch (17.48 ± 0.86) treatment (Figure 9) as the nutrient supplementation was able to prevent the substrate inhibition within the fermentation media, elevating the erythritol yield (Hung et al., 2023). Therefore, it was preferable to the other treatments in terms of cost-effectiveness. This aligns with the research by Oh et al. (2001), where the fed-batch production system exhibits a higher erythritol production, in which the volumetric production rate is increased up to 58%, compared to the batch fermentation system. In addition to that, Rywińska et al. (2024a) experimented with the fed-batch fermentation was proven to improve the erythritol yield from 71.3 g/L to 97.5 g/L, compared to the batch treatment using *Y. lipolytica* Wratislavia K1 strain (UV-mutated). The first treatment is the supplementation of molasses, with the absence of yeast extract. This has been hypothesized due to the molasses as a primary substrate, containing a high amount of sucrose that can be hydrolyzed into glucose and fructose, playing a role in the pentose phosphate pathway, which the availability will be able to increase the reaction efficiency and accelerate the erythritol production (Hawaz et al., 2024; Sharkey, 2021). Molasses, as a carbon source itself, already consists of a few nitrogen compounds that help the fermentation process (Seshadrinathan and Chakraborty, 2022). On the other hand, based on the findings by Rojo et al. (2023), nitrogen sources are important for supporting the metabolism and the fermentation of the yeast, yet it is not directly converted into erythritol. According to the research by Geremew Kassa et al. (2024), molasses contains calcium content. Therefore, a high molasses content might lead to the restriction of the invertase enzyme activity, which is a critical enzyme for the erythritol conversion pathway. Nevertheless, a suitable concentration of molasses should be taken into account to enhance erythritol production, as the other treatments with higher nutrient supplementation exhibit less production of erythritol. Aside from that, the addition of molasses/yeast extract during the feeding process was only around 2% (v/v), and has been assumed to have no significant effect, therefore dilution effect is not taken into account.

**TABLE 4:** Erythritol Concentration (g/L), Erythritol Yield (g/g), and Erythritol Productivity (g/Lh) on batch and fed-batch fermentation systems.

Fermentation system	Erythritol concentration (g/L)	Erythritol yield mass (g/g)	Erythritol volumetric productivity (g/Lh)	Comparison to control (*p*-value)
Batch	17.48 ± 0.86	0.262 ± 0.00	0.095 ± 0.021	Control
Treatment 1 Fed-batch	26.52 ± 1.61	0.501 ± 0.032	0.158 ± 0.01	*(increase)
Treatment 2 Fed-batch	22.80 ± 0.13	0.318 ± 0.065	0.136 ± 0.001	*(increase)
Treatment 4 Fed-batch	23.17 ± 0.55	0.351 ± 0.013	0.138 ± 0.003	*(increase)
Treatment 4 Fed-batch	23.94 ± 0.84	0.334 ± 0.018	0.142 ± 0.005	*(increase)

Note: “*” indicates a significant difference (*p* ≤ 0.05) toward the experimental controls.

**FIGURE 10:**
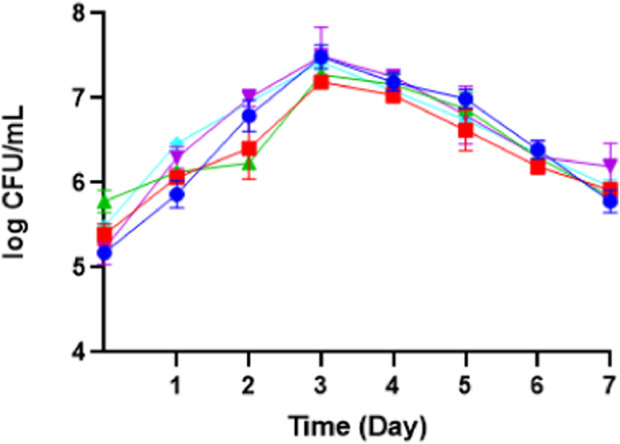
The growth phase of *M. pollinis* SP5 using batch (turquoise) and fed-batch fermentation systems with different feeding treatments: treatment 1 (blue), treatment 2 (red), treatment 3 (green), and treatment 4 (purple) during 7 days of fermentation.

Determining the colony forming unit (CFU), dry cell weight, and pH is beneficial for observing the growth analysis of *M. pollinis* and whether the erythritol production grows linearly with the yeast growth. The biomass growth via dry cell weight measurement, which the trend exhibits, increases on the third day as the exponential growth period while continuing the stationary growth and eventually ending the growth cycle on the seventh day of incubation. Similar to the biomass trend, the log CFU/mL exhibited a pattern of the cell growing exponentially on the third day and continued to slightly declined on the fifth to seventh days. (See Figure 10) (Supplementary Figure S6). Even so, several biomass concentrations fluctuate on the second and third day, suggesting that the addition of substrate influenced yeast growth. The strong correlation shown has elucidated that as the cell viability increases, the biomass also increases, indicating the cells were in healthy and suitable metabolic conditions (Ughy et al., 2023). On the other hand, the culture pH for all of the treatments was constant. It all started at around pH 5. During the cultivation, the pH of each treatment was slightly dropped to about 4.5, which then continued static, until the seventh day of incubation (Supplementary Figure S7).

## Conclusion

Erythritol can be produced using biotechnological methods such as fermentation using *Moniliella pollinis* SP5 as a cell factory. This study also highlights molasses as a cost-effective carbon source for erythritol production, which supports the bioeconomic as an agroindustrial byproduct utilization. This study involves the optimization of several variables to elevate erythritol production, incorporating the substrate concentration ratio, NaCl concentration, pH, and mode of operation (batch and fed-batch). Using OFAT, the variables were tested one by one, which resulted in the most optimal media of 200 g/L of molasses, 7 g/L of yeast extract, 25 g/L of NaCl, pH of 5, and using a fed-batch mode (25 g/L of molasses on the second and third day). This combination produces 26.52 ± 1.61 g/L of erythritol concentration or about 51% increase compared to the control, 0.262 ± 0.00 g/g of yield mass, and 0.095 ± 0.021 g/Lh of volumetric productivity, significantly enhanced the erythritol production. Continuing this experiment, further product yield and purity optimization should be examined through purification in downstream processing, elevating the economic viability for erythritol production.

## Data Availability

The original contributions presented in the study are included in the article/Supplementary Material, further inquiries can be directed to the corresponding author.

## References

[B1] Anzola-Rojas M. Gonçalves da Fonseca S. Canedo da Silva C. Maia de Oliveira V. Zaiat M. Zaiat M. (2015). The use of the carbon/nitrogen ratio and specific organic loading rate as tools for improving biohydrogen production in fixed-bed reactors. Biotechnol. Rep. 5, 46–54. 10.1016/j.btre.2014.10.010 PMC5466190 28626682

[B43] Bellou S. Makri A. Triantaphyllidou I.-E. Papanikolaou S. Aggelis G. (2014). Morphological and metabolic shifts of Yarrowia lipolytica induced by alteration of the dissolved oxygen concentration in the growth environment. Microbiology 160, 807–817. 10.1099/mic.0.074302-0 24509502

[B2] Biggelaar L. J. C. J. d. Eussen S. J. Sep S. J. Mari A. Ferrannini E. Dongen M. C. J. M. v. (2017). Associations of dietary glucose, fructose, and sucrose with β-cell function, insulin sensitivity, and type 2 diabetes in the maastricht study. Nutrients 9 (4), 380. 10.3390/nu9040380 28406435 PMC5409719

[B3] Carly F. Fickers P. (2018). Erythritol production by yeasts: a snapshot of current knowledge. Yeast 35 (7), 455–463. 10.1002/yea.3306 29322598

[B4] Chen C.-C. Bates R. Carlson J. (2015). Effect of environmental and cultural conditions on medium pH and explant growth performance of Douglas-fir (*Pseudotsuga menziesii*) shoot cultures. F1000 Res. 3, 298. 10.12688/f1000research.5919.2 PMC4617322 26535110

[B5] Daza-Serna L. Serna-Loaiza S. Masi A. Mach R. L. Mach-Aigner A. R. Friedl A. (2021). From the culture broth to the erythritol crystals: an opportunity for circular economy. Appl. Microbiol. Biotechnol. 105 (11), 4467–4486. 10.1007/s00253-021-11355-2 34043080 PMC8195806

[B44] Deshpande M. S. Kulkarni P. P. Kumbhar P. S. Ghosalkar A. R. (2022). Erythritol production from sugar based feedstocks by *Moniliella pollinis* using lysate of recycled cells as nutrients source. Process Biochem. 112, 45–52. 10.1016/j.procbio.2021.11.020

[B6] De Vasconcelos J. N. (2015). Chapter 15: ethanol fermentation. Sugarcane, 311–340. 10.1016/b978-0-12-802239-9.00015-3

[B7] Dupont S. Beney L. Ritt J.-F. Lherminier J. Gervais P. (2010). Lateral reorganization of plasma membrane is involved in the yeast resistance to severe dehydration. Biochimica Biophysica Acta (BBA) - Biomembr. 1798 (5), 975–985. 10.1016/j.bbamem.2010.01.015 20116363

[B8] Fickers P. Cheng H. Sze Ki Lin C. (2020). Sugar alcohols and organic acids synthesis in Yarrowia lipolytica: where are we? Microorganisms 8 (4), 574. 10.3390/microorganisms8040574 32326622 PMC7232202

[B9] Geremew Kassa M. Asemu A. M. Belachew M. T. Satheesh N. Abera B. D. Alemu Teferi D. (2024). Review on the application, health usage, and negative effects of molasses. CyTA J. Food 22 (1). 10.1080/19476337.2024.2321984

[B10] Ghezelbash G. R. Nahvi I. Rabbani M. (2012). Study of polyols production by *Yarrowia lipolytica* in batch culture and optimization of growth condition for maximum production. Jundishapur J. Microbiol. 5 (4), 546–549. 10.5812/jjm.3524

[B11] Guyot S. Pottier L. Bertheau L. Dumont J. Dorelle Hondjuila Miokono E. Dupont S. (2021). Increased xerotolerance of *Saccharomyces cerevisiae* during an osmotic pressure ramp over several generations. Microb. Biotechnol. 14 (4), 1445–1461. 10.1111/1751-7915.13789 33739621 PMC8313259

[B12] Hawaz E. Tafesse M. Tesfaye A. Kiros S. Beyene D. Kebede G. (2024). Bioethanol production from sugarcane molasses by co-fermentation of *Saccharomyces cerevisiae* isolate TA2 and *Wickerhamomyces anomalus* isolate HCJ2F-19. Ann. Microbiol. 74 (1), 13. 10.1186/s13213-024-01757-8

[B13] Hijosa-Valsero M. Paniagua-García A. I. Díez-Antolínez R. (2022). Cell immobilization for erythritol production. J. Fungi 8 (12), 1286. 10.3390/jof8121286 PMC9785647 36547619

[B14] Hung Y.-H. Chae M. Sauvageau D. Bressler D. (2023). Adapted feeding strategies in fed-batch fermentation improve sugar delivery and ethanol productivity. Bioengineered. 14, 2250950. 10.1080//21655979.2023.2250950 37655550 PMC10478740

[B15] Kar M. Chourasiya Y. Maheshwari R. Tekade R. K. (2019). Chapter 2-Current developments in excipient science: implication of quantitative selection of each excipient in product development. Sci. Acad. Press. Available at: https://www.sciencedirect.com/science/article/abs/pii/B9780128179093000029.

[B16] Khatape A. B. Rangaswamy V. Dastager S. G. (2023). Strain improvement for enhanced erythritol production by *Moniliella pollinis* Mutant-58 using jaggery as a cost-effective substrate. Int. Microbiol. 27 (2), 581–596. 10.1007/s10123-023-00411-8 37525085

[B17] Li F. Xiong X. S. Yang Y. Y. Wang J. J. Wang M. M. Tang J. W. (2021). Effects of NaCl concentrations on growth patterns, phenotypes associated with virulence, and energy metabolism in *Escherichia coli* BW25113. Front. Microbiol. 12, 705326. 10.3389/fmicb.2021.705326 34484145 PMC8415458

[B18] Li Z. Lou Y. Ding J. Liu B.-F. Xie G.-J. Ren N.-Q. (2020). Metabolic regulation of ethanol-type fermentation of anaerobic acidogenesis at different pH based on transcriptome analysis of *Ethanoligenens harbinense* . Biotechnol. Biofuels 13 (1), 101. 10.1186/s13068-020-01740-w 32518589 PMC7268672

[B19] Liu X. Lv J. Xu J. Xia J. He A. Zhang T. (2018). Effects of osmotic pressure and pH on citric acid and erythritol production from waste cooking oil by *Yarrowia lipolytica* . Eng. Life Sci. 18 (6), 344–352. 10.1002/elsc.201700114 32624914 PMC6999400

[B20] Liu X. Yu X. He A. Xia J. He J. Deng Y. (2022). One-pot fermentation for erythritol production from distillers grains by the co-cultivation of *Yarrowia lipolytica* and *Trichoderma reesei* . Bioresour. Technol. 351, 127053. 10.1016/j.biortech.2022.127053 35337991

[B21] Lund P. A. De Biase D. Liran O. Scheler O. Mira N. P. Cetecioglu Z. (2020). Understanding how microorganisms respond to acid pH is central to their control and successful exploitation. Front. Microbiol. 11, 556140. 10.3389/fmicb.2020.556140 33117305 PMC7553086

[B22] Malairuang K. Krajang M. Sukna J. Rattanapradit K. Chamsart S. (2020). High cell density cultivation of *Saccharomyces cerevisiae* with intensive multiple sequential batches together with a novel technique of fed-batch at cell level (FBC). Processes 8 (10), 1321. 10.3390/pr8101321

[B23] Mazi T. A. Stanhope K. L. (2023). Erythritol: an in-depth discussion of its potential to be a beneficial dietary component. Nutrients 15 (1), 204. 10.3390/nu15010204 36615861 PMC9824470

[B24] Mirończuk A. M. Dobrowolski A. Rakicka M. Rywińska A. Rymowicz W. (2015). Newly isolated mutant of *Yarrowia lipolytica* MK1 as a proper host for efficient erythritol biosynthesis from glycerol. Process Biochem. 50 (1), 61–68. 10.1016/j.procbio.2014.10.020

[B25] Mohd-Zaki Z. Bastidas-Oyanedel J. Lu Y. Hoelzle R. Pratt S. Slater F. (2016). Influence of pH regulation mode in glucose fermentation on product selection and process stability. Microorganisms 4 (1), 2. 10.3390/microorganisms4010002 27681895 PMC5029507

[B26] Oh D.-K. Cho C.-H. Lee J.-K. Kim S.-Y. (2001). Increased erythritol production in fed-batch cultures of *Torula* sp. by controlling glucose concentration. J. Industrial Microbiol. Biotechnol. 26 (4), 248–252. 10.1038/sj.jim.7000122 11464275

[B27] Ortiz S. R. Heinz A. Hiller K. Field M. S. (2022). Erythritol synthesis is elevated in response to oxidative stress and regulated by the non-oxidative pentose phosphate pathway in A549 cells. Front. Nutr. 9, 953056. 10.3389/fnut.2022.953056 36276829 PMC9582529

[B28] Poontawee R. Limtong S. (2020). Feeding strategies of two-stage fed-batch cultivation processes for microbial lipid production from sugarcane top hydrolysate and crude glycerol by the oleaginous red yeast *Rhodosporidiobolus fluvialis* . Microorganisms 8 (2), 151. 10.3390/microorganisms8020151 31979035 PMC7074793

[B45] Rakicka-Pustułka M. Mirończuk A. M. Celińska E. Białas W. Rymowicz W. (2020). Scale-up of the erythritol production technology – process simulation and techno-economic analysis. J. Clean. Prod. 257, 120533. 10.1016/j.jclepro.2020.120533

[B29] Rojo M. C. Talia P. M. Lerena M. C. Ponsone M. L. Gonzalez M. L. Becerra L. M. (2023). Evaluation of different nitrogen sources on growth and fermentation performance for enhancing ethanol production by wine yeasts. Heliyon 9 (12), e22608. 10.1016/j.heliyon.2023.e22608 38213578 PMC10782155

[B30] Rywińska A. Tomaszewska-Hetman L. Juszczyk P. Rakicka-Pustułka M. Bogusz A. Rymowicz W. (2024a). Enhanced production of erythritol from glucose by the newly obtained uv mutant *Yarrowia lipolytica* K1UV15. Molecules 29 (10), 2187. 10.3390/molecules29102187 38792051 PMC11124037

[B31] Rywińska A. Tomaszewska-Hetman L. Lazar Z. Juszczyk P. Sałata P. Malek K. (2024b). Application of new *Yarrowia lipolytica* transformants in production of citrates and erythritol from glycerol. Int. J. Mol. Sci. 25 (3), 1475. 10.3390/ijms25031475 38338753 PMC10855631

[B32] Rzechonek D. A. Dobrowolski A. Rymowicz W. Mirończuk A. M. (2018). Recent advances in biological production of erythritol. Crit. Rev. Biotechnol. 38 (4), 620–633. 10.1080/07388551.2017.1380598 28954540

[B33] Seshadrinathan S. Chakraborty S. (2022). Fermentative production of erythritol from cane molasses using *Candida magnoliae*: media optimization, purification, and characterization. Sustainability 14 (16), 10342. 10.3390/su141610342

[B34] Sharkey T. D. (2021). Pentose phosphate pathway reactions in photosynthesizing cells. Cells 10 (6), 1547. 10.3390/cells10061547 34207480 PMC8234502

[B35] Skirvin R. M. Chu M. C. Mann M. L. Young H. Sullivan J. Fermanian T. (1986). Stability of tissue culture medium pH as a function of autoclaving, time, and cultured plant material. Plant Cell Rep. 5 (4), 292–294. 10.1007/bf00269825 24248250

[B36] Sun H. Saeedi P. Karuranga S. Pinkepank M. Ogurtsova K. Duncan B. B. (2022). IDF diabetes Atlas: global, regional and country-level diabetes prevalence estimates for 2021 and projections for 2045. Diabetes Res. Clin. Pract. 183 (109119), 109119. 10.1016/j.diabres.2021.109119 34879977 PMC11057359

[B37] Tomaszewska L. Rywińska A. Rymowicz W. (2014). High selectivity of erythritol production from glycerol by *Yarrowia lipolytica* . Biomass Bioenergy 64, 309–320. 10.1016/j.biombioe.2014.03.005

[B38] Ughy B. Nagyapati S. Lajko D. B. Letoha T. Prohaszka A. Deeb D. (2023). Reconsidering dogmas about the growth of bacterial populations. Cells 12 (10), 1430. 10.3390/cells12101430 37408264 PMC10217356

[B39] Wang S. Tian R. Liu B. Wang H. Liu J. Li C. (2021). Effects of carbon concentration, oxygen, and controlled pH on the engineering strain *Lactiplantibacillus casei* E1 in the production of bioethanol from sugarcane molasses. Amb. Express 11 (1), 95. 10.1186/s13568-021-01257-x 34176008 PMC8236424

[B40] Yang L.-B. Dai X.-M. Zheng Z.-Y. Zhu L. Zhan X.-B. Lin C.-C. (2015). Proteomic analysis of erythritol-producing *Yarrowia lipolytica* from glycerol in response to osmotic pressure. J. Microbiol. Biotechnol. 25 (7), 1056–1069. 10.4014/jmb.1412.12026 25737116

[B41] Yang L. B. Zhan X. B. Zheng Z. Y. Wu J. R. Gao M. J. Lin C. C. (2014). A novel osmotic pressure control fed-batch fermentation strategy for improvement of erythritol production by *Yarrowia lipolytica* from glycerol. Bioresour. Technol. 151, 120–127. 10.1016/j.biortech.2013.10.031 24215768

[B42] Yang S. Li Y. Guo B. You J. Zhang X. Shao M. (2024). Comparative transcriptomics analysis-guided metabolic engineering of *Yarrowia lipolytica* for improved erythritol and fructooligosaccharides production. Bioresour. Technol. 408, 131188. 10.1016/j.biortech.2024.131188 39089656

