# Supplementary material for: Optimization of erythritol production through fermentation using molasses as carbon source
Source: Acta Biochim Pol. 2025 Jan 8;71:14000. doi: 10.3389/abp.2024.14000 (PMC11750574; doi:10.3389/abp.2024.14000)
Supplement: Supplementary file 1 [file DataSheet1.PDF]

Supplementary Information

Optimization of Erythritol Production through Fermentation Using Molasses as Carbon Source

Riahna Kembaren[1]*, Arli Aditya Parikesit[1], Jocelyn Nataniel[1], Nethania Angeline Dharmawan[1], Priscilla Angelique[1], Charlivo Mikaichi Dungus[1], Solmaz Aslanzadeh[1]

[1] Indonesia International Institute for Life Sciences, Department of Biotechnology, 13210 Pulo Gadung, East Jakarta, Indonesia

S1. pH of fermentation media of molasses-to-yeast extract concentration ratio optimization

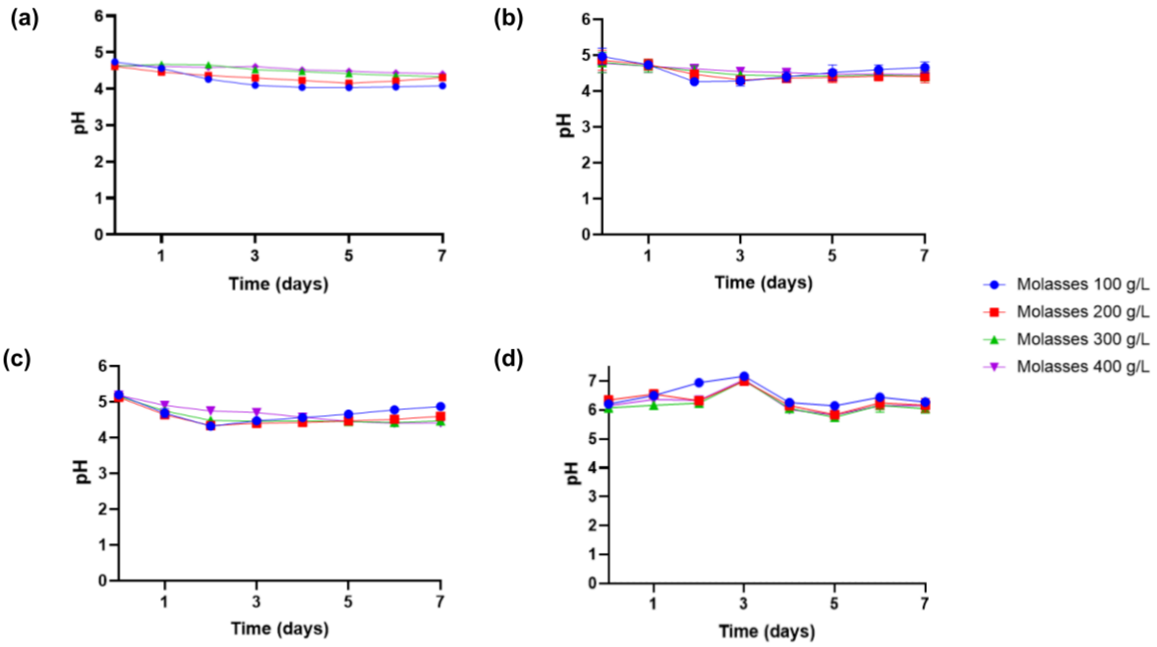

Figure S1. pH of fermentation media of molasses/yeast extract ratio optimization using 100, 200, 300, and 400 g/L molasses. (a) Media combination of 1 g/L yeast; (b) Media combination of 4 g/L yeast; (c) Media combination of 7 g/L yeast; and (d) Media combination of 10 g/L yeast.

S2. Dry cell weight (g/L) of molasses-to-yeast extract ratio optimization

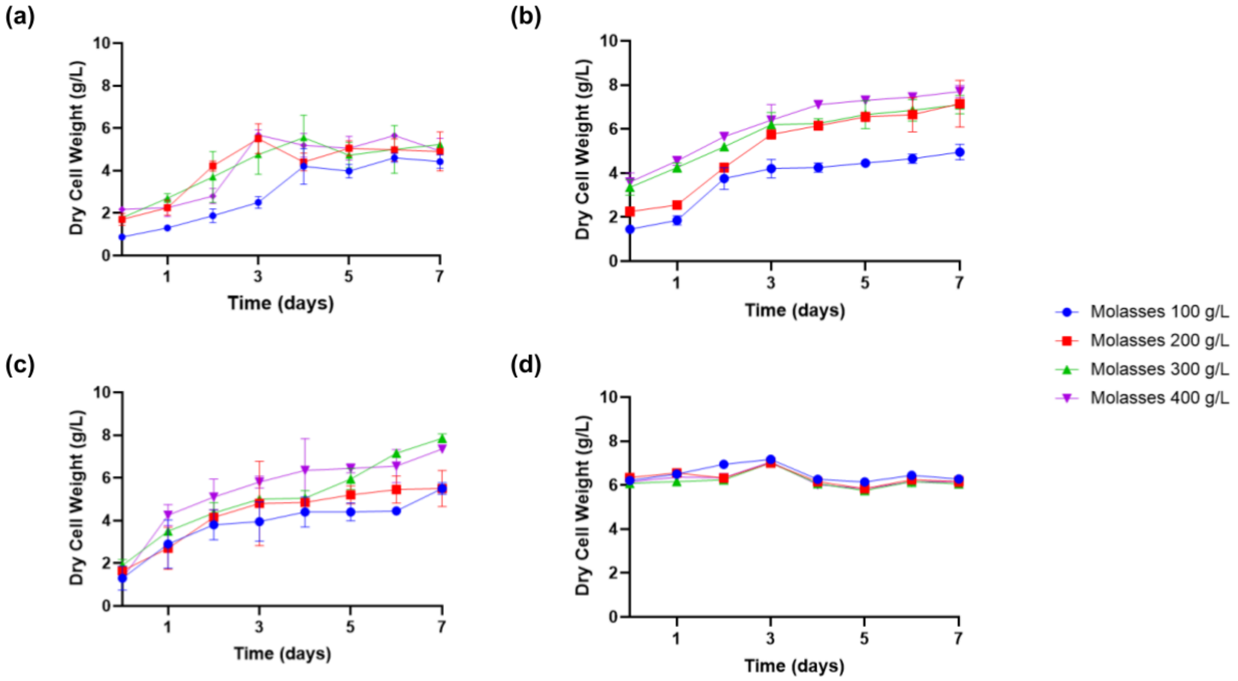

Figure S2. Dry weight cell of molasses/yeast Ratio optimization using 100, 200, 300, and 400 g/L molasses. (a) Media combination of 1 g/L yeast; (b) Media combination of 4 g/L yeast; (c) Media combination of 7 g/L yeast; and(d) Media combination of 10 g/L yeast.

S3. Dry cell weight (g/L) of NaCl optimization

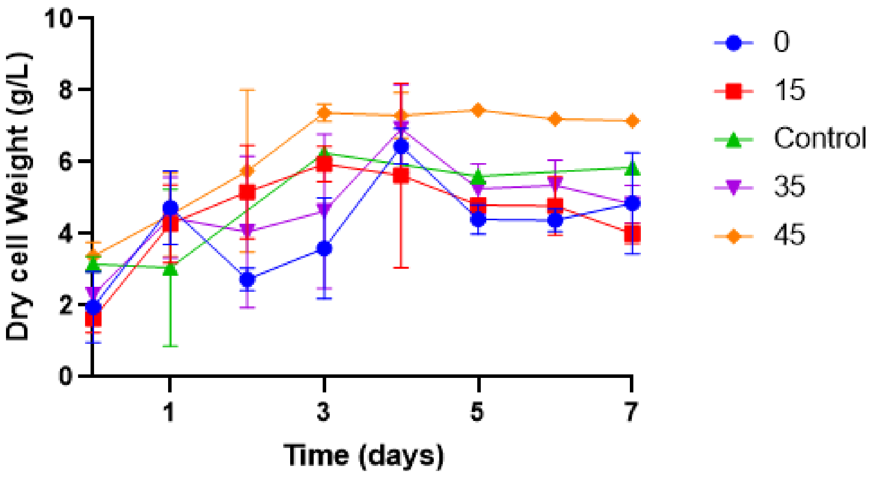

Figure S3. Dry cell weight of optimization NaCl (0, 15, 25, 35, & 45 g/L) from 7 days of incubation

S4. pH of fermentation media from NaCl optimization

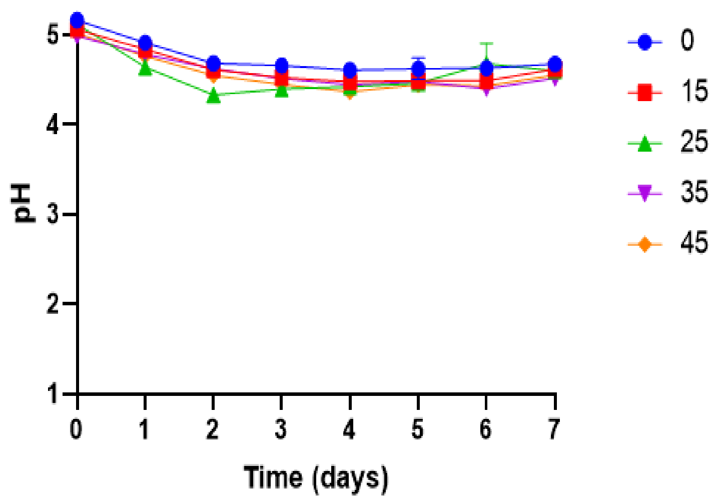

Figure S4. pH of fermentation media from NaCl optimization for 7 days of incubation using different NaCl concentrations: 0, 15, 25, 35, and 45 g/L.

S5. Dry cell weight (g/L) analysis of pH optimization

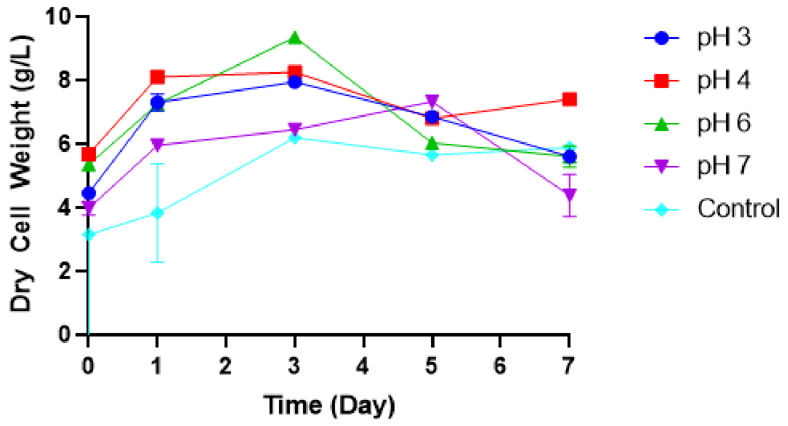

Figure S5. Dry weight cell for 7 days of incubation on different initial pH of fermentation media: pH 3, 4, 5 (control), 6, and 7.

S6. Dry cell weight (g/L) of fed-batch optimization

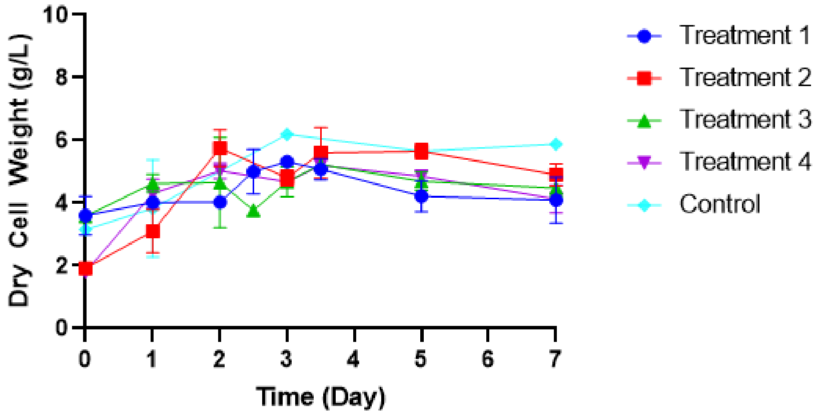

Figure S6. Dry weight cell of a fed-batch mode of operation optimization using supplementation. Treatment 1: Add 25 g/L of molasses on day-2 and day-3. Treatment 2: Add 50 g/L of molasses on day 3. Treatment 3: Add 25 g/L of molasses and 0.875 g/L of yeast extract on the second and third days. Treatment 4: Add 50 g/L of molasses and 1.75 g/L of yeast extract.

S7. pH of fermentation of fed-batch optimization

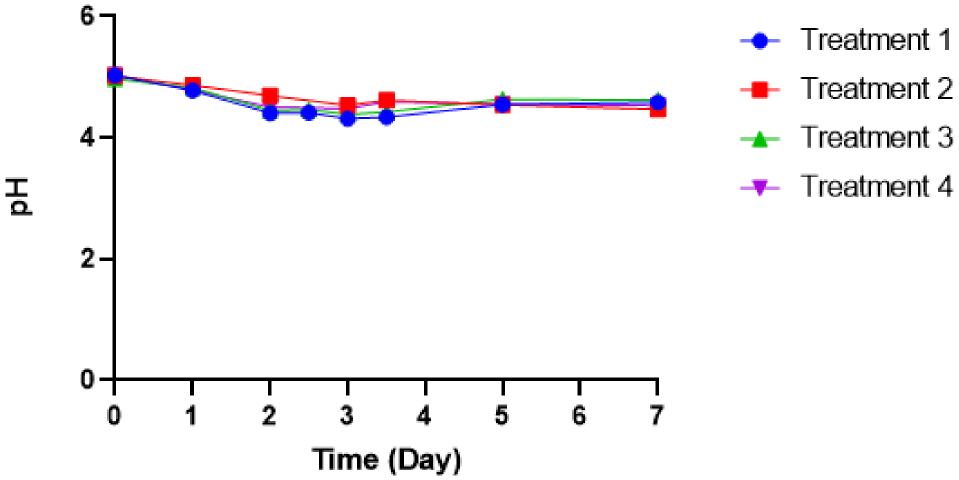

Figure S7. pH of a fed-batch mode of operation optimization using supplementation. Treatment 1: Add 25 g/L of molasses on day-2 and day-3. Treatment 2: Add 50 g/L of molasses on day 3. Treatment 3: Add 25 g/L of molasses and 0.875 g/L of yeast extract on the second and third days. Treatment 4: Add 50 g/L of molasses and 1.75 g/L of yeast extract.

Table S1. Statistical analysis of student t-test results of different molasses-to-yeast extract treatments in fermentation compared to the control (n=2)

**Molasses/Yeast extract concentration**	**p-value**	**t, df**	**Difference between means ± SEM**	**Significantly different (P<0.05)**
100/1	0.0041	t=15.54, df=2	6.546 ± 0.4213	Yes (**)
300/1	0.0643	t=3.751, df=2	2.381 ± 0.6347	ns
400/1	0.0080	t=11.14, df=2	6.269 ± 0.5630	Yes (**)
100/4	0.0069	t=11.99, df=2	5.303 ± 0.4422	Yes (**)
200/4	0.2751	t=1.488, df=2	1.650 ± 1.109	ns
300/4	0.2944	t=1.408, df=2	0.8990 ± 0.6384	ns
400/4	0.0838	t=3.234, df=2	1.108 ± 0.3427	ns
100/7	0.0135	t=8.517, df=2	4.719 ± 0.5541	Yes (*)
200/7	0.0236	t=6.391, df=2	3.884 ± 0.6077	Yes (*)
300/7	0.4168	t=1.015, df=2	1.236 ± 1.217	ns
400/7	0.0203	t=6.918, df=2	4.933 ± 0.7130	Yes (*)
100/10	0.0161	t=7.785, df=2	7.944 ± 1.020	Yes (*)
200/10	0.0279	t=5.861, df=2	7.419 ± 1.266	Yes (*)
300/10	0.0918	t=3.069, df=2	6.114 ± 1.992	ns
400/10	0.0510	t=4.257, df=2	5.389 ± 1.266	ns

Table S2. Statistical analysis of student t-test results of different NaCl treatments in fermentation compared to the control (n=2)

Treatment	p-value	t, df	Difference between means ± SEM	Significantly different (P<0.05)
0 g/L NaCl	0.1310	t=2.846, df=2	4.820 ± 1.941	ns
15 g/L NaCl	0.1012	t=2.899, df=2	4.875 ± 1.682	ns
35 g/L NaCl	0.0930	t=3.045, df=2	6.240 ± 2.049	ns
45 g/L NaCl	0.1045	t=2.846, df=2	5.170 ± 1.816	ns

Table S3. Statistical analysis of student t-test results of different pH treatments in fermentation compared to the control (n=2)

Treatment	p-value	t, df	Difference between means ± SEM	Significantly different (P<0.05)
pH 3	0.0224	t=6.573, df=2	10.90 ± 1.659	Yes (*)
pH 4	0.0210	t=6.798, df=2	9.692 ± 1.426	Yes (*)
pH 6	0.0111	t=9.409, df=2	8.092 ± 0.8600	Yes (*)
pH 7	0.0089	t=10.51, df=2	9.120 ± 0.8680	Yes (*)

Table S4. Statistical analysis of student t-test result of fed-batch mode of operation compared to the control (n=2)

Treatment	p-value	t, df	Difference between means ± SEM	Significantly different (P<0.05)
Treatment 1	0.0239	t=6.348, df=2	9.049 ± 1.425	Yes (*)
Treatment 2	0.0254	t=6.160, df=2	5.327 ± 0.8647	Yes (*)
Treatment 3	0.0264	t=6.030, df=2	5.690 ± 0.9437	Yes (*)
Treatment 4	0.0252	t=6.177, df=2	6.459 ± 1.046	Yes (*)
